# Informative RNA base embedding for RNA structural alignment and clustering by deep representation learning

**DOI:** 10.1093/nargab/lqac012

**Published:** 2022-02-22

**Authors:** Manato Akiyama, Yasubumi Sakakibara

**Affiliations:** Department of Biosciences and Informatics, Keio University, 223-8522, Japan; Department of Biosciences and Informatics, Keio University, 223-8522, Japan

## Abstract

Effective embedding is actively conducted by applying deep learning to biomolecular information. Obtaining better embeddings enhances the quality of downstream analyses, such as DNA sequence motif detection and protein function prediction. In this study, we adopt a pre-training algorithm for the effective embedding of RNA bases to acquire semantically rich representations and apply this algorithm to two fundamental RNA sequence problems: structural alignment and clustering. By using the pre-training algorithm to embed the four bases of RNA in a position-dependent manner using a large number of RNA sequences from various RNA families, a context-sensitive embedding representation is obtained. As a result, not only base information but also secondary structure and context information of RNA sequences are embedded for each base. We call this ‘informative base embedding’ and use it to achieve accuracies superior to those of existing state-of-the-art methods on RNA structural alignment and RNA family clustering tasks. Furthermore, upon performing RNA sequence alignment by combining this informative base embedding with a simple Needleman–Wunsch alignment algorithm, we succeed in calculating structural alignments with a time complexity of *O*(*n*^2^) instead of the *O*(*n*^6^) time complexity of the naive implementation of Sankoff-style algorithm for input RNA sequence of length *n*.

## INTRODUCTION

**Figure 1.**
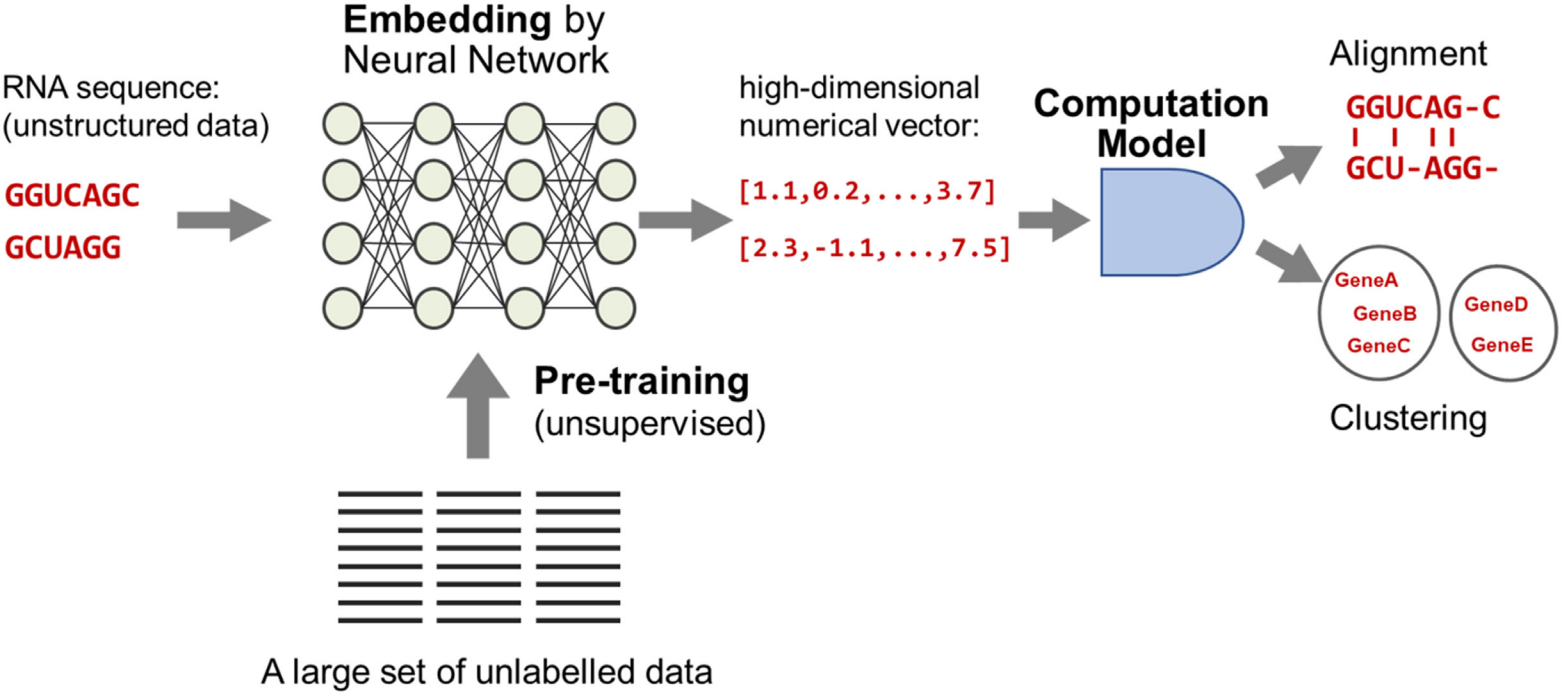
Schematic view of the pre-training-based embedding and its downstream analysis. The pre-trained neural network with a large set of unlabelled data encodes input DNA sequences into high-dimensional numerical vectors. The embedding by pre-trained neural networks is effective for downstream analysis such as DNA sequence alignment and clustering.

Unstructured data, such as biological sequences and networks, require an embedding operation that encodes the unstructured data into a high-dimensional numerical vector space. This is a necessary step for processing unstructured data in downstream analysis using computational models such as neural networks. In the deep learning field, embedding using the pre-training framework with a large set of unlabelled data has been shown to be effective for the downstream supervised learning task even when smaller size of labelled data is available. When embedding an RNA sequence, each nucleotide (A, C, G, U) is usually encoded to a numerical representation so that the RNA sequence is embedded into a numerical vector. An effective embedding method further attempts to encode contextual information into the numerical vector representation (see Figure 1).

Recently, DNA, RNA and amino acid sequences have been attempted to be effectively embedded using deep representation learning, especially techniques developed in the field of natural language processing (1–3). These studies are based on the idea that nucleotide composition and sequence structure determine the motif and function of a gene sequence, just as the complex grammatical structure of natural language determines the meaning of a sentence. As a consequence, word embedding techniques for natural language have been applied to nucleotides for DNA sequences. In the dna2vec method (4), word2vec is applied to a DNA sequence to obtain the distributed representation of *k*-mers (a DNA subsequence of length *k*). Word2vec, an effective word embedding technique (5) that vectorizes the context and meaning of a word using a large amount of text data, is based on the hypothesis that words with similar meanings have similar peripheral words. Dna2vec adopts the word2vec technique by defining a *k*-mer as a word in the DNA sequence; however, since dna2vec assumes a sufficient number of different words used for embedding, the four nucleotides (four words) are not large enough to obtain an effective embedding when dna2vec is applied to base-by-base DNA sequence embedding.

Two recently developed state-of-the-art embedding methods, namely, embeddings from language models (ELMo) and bidirectional encoder representations from transformers (BERT), are used to generate context-sensitive distributed word representations (6,7). In these methods, the same word is assigned to different distributed representations depending on the context. In particular, BERT is a pre-training algorithm that obtains word and sentence embeddings by performing two tasks: a masked language modelling (MLM) task and a next sentence prediction (NSP) task. The MLM task predicts multiple masked tokens (words) in a sentence, whereas the NSP task determines whether two statements are consecutive. UniRep (8) and PLUS (9) are representative examples of applying BERT to protein sequence representation; specifically, UniRep obtains the embedding of each amino acid in a protein sequence and uses this embedding to achieve accurate structural and functional predictions of proteins.

In this study, we propose RNABERT for the effective embedding of RNA bases by adopting the pre-training BERT algorithm to non-coding RNA (ncRNA). We apply informative base embedding to encode the characteristics of each RNA family and structure. To see whether this informative base embedding technique successfully captures these characteristics, we apply RNABERT to two basic RNA sequence analysis tasks: structural alignment and clustering. Then, we evaluate the quality of the informative base embedding results by structural alignment of RNA sequences and by RNA family clustering.

The first important problem in RNA sequence analysis is the structural alignment of RNA sequences, which calculates the alignment of not only RNA sequences but also their secondary structures. The most influential method for the structural alignment of RNA sequences is the Sankoff algorithm, which simultaneously performs secondary structure prediction and alignment (10). However, the time complexity of the naive implementation of the Sankoff algorithm is *O*(*n*^6^) for the length *n* of input RNA sequences, and accelerating the Sankoff algorithm is an unsolved hard problem (11). While Sankoff-style algorithms such as LocARNA (12) and Dynalign (13) calculate the alignment considering the secondary structure, a standard sequence-based (non-structural) alignment method such as the Needleman–Wunsch algorithm (14) determines only the correspondence between each base position of two input sequences, and its time complexity is only *O*(*n*^2^) using the dynamic programming technique. Hence, we aim to apply the informative base embedding to determine the position-dependent and secondary structure-dependent score matrix in calculating alignments so that the structural alignment is obtained using a simple Needleman–Wunsch algorithm instead of the computationally expensive Sankoff-style algorithm.

Building an appropriate clustering algorithm for ncRNAs is an effective step towards unsupervised analysis of ncRNA sequences without their family labels (15,16), as high-throughput sequencing continues to generate a large number of RNA sequences, including novel transcripts. With the recent increase in deep learning usage, many algorithms for ncRNA classification (supervised clustering) using convolutional neural networks (CNNs) and recurrent neural networks (RNNs) have been proposed (17,18). These algorithms adopt a simple embedding technique, one-hot encoding of RNA bases. Most of these algorithms utilize supervised learning using ncRNA families as labels for training. Nevertheless, since supervised learning requires the data to be labelled, this approach is not practical when analysing ncRNA sequences without their family labels.

For our goals of RNA structural alignment of lower computational complexity and accurate RNA family clustering, we construct an informative base embedding method, RNABERT, for RNA sequences that takes into account the context and secondary structure of RNA sequences through two training tasks: MLM and structural alignment learning (SAL). In RNABERT, pre-training is performed using a large number of unlabelled ncRNA sequences. RNABERT introduces a novel pre-training task, SAL, in addition to the usual MLM task to more explicitly incorporate RNA secondary structure information into the base embedding for structural alignments. The SAL task employs pre-training using seed alignments obtained from the Rfam database (19) so that the bases aligned in the seed structural alignment are expected to have more similar embeddings. By alternately training the MLM and SAL tasks, RNA base embedding can be expected to adequately capture the structural differences among RNA families. We compare the accuracy and computational complexity of structural alignment and family clustering of RNA sequences between our method and the state-of-the-art methods.

## MATERIALS AND METHODS

### The architecture of the RNABERT model

**Figure 2.**
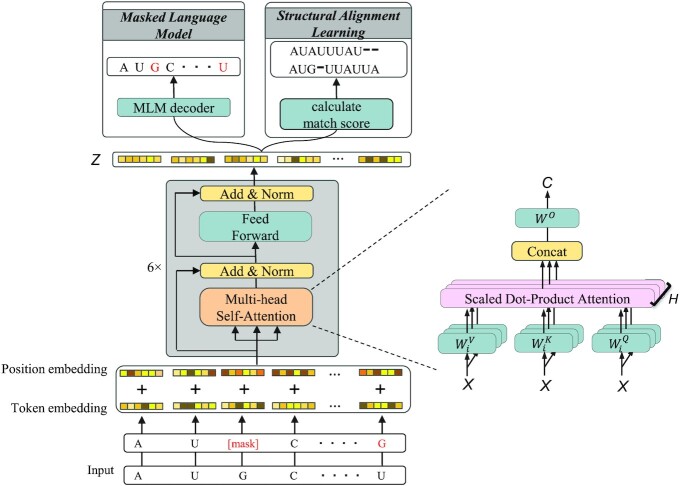
Architecture of the RNABERT model. The RNABERT model consists of three components: token and position embedding, a transformer layer and pre-training tasks. Token and position embedding randomly generates a 120-dimensional vector representing four RNA bases. The transformer layer component consists of a stack of six transformer layers, each of which is composed of a multi-head self-attention mechanism followed by a feedforward neural network. The final output from the transformer layer is an informative base embedding, denoted *Z*. The weights of the transformer layer are trained by alternately training two different tasks (MLM and SAL) on top of the output of the transformer layer.

The architecture of the RNABERT model consists of three components: token and position embedding, a transformer layer and pre-training tasks. The input to RNABERT is an RNA sequence. First, the token embedding randomly generates a 120-dimensional numerical vector that encodes four RNA bases (A, C, G, U) and assigns the same vector to each base in the input RNA sequence. Second, the position embedding generates a 120-dimensional vector that encodes the position information of each base in the input RNA sequence. Third, the element-wise sum of token embedding and position embedding for each base in the input RNA sequence is fed to the transformer layer. The transformer layer component consists of a stack of six transformer layers, each of which is composed of a multi-head self-attention mechanism followed by a feedforward neural network. The final output from the transformer layer is an informative base embedding, denoted $Z$. The weight parameters of the transformer layer are trained by alternately training two different tasks (MLM and SAL) on top of the output of the transformer layer. The architecture of RNABERT is illustrated in Figure 2.

The self-attention mechanism (20) is a central component of the transformer layer. For the transformer layer that takes the output of the previous layer $X\ = \ [ {{x_1}, \ldots ,{x_n}} ]$ as input, the multi-head self-attention mechanism with $H$ heads computes the output sequence $C\ = \ [ {{c_1}, \ldots ,{c_n}} ]$ with the following formula:

$$\begin{equation*}C\ = \ Concat\left( {hea{d_1}, \ldots ,hea{d_H}} \right){W^O},\end{equation*}$$

$$\begin{equation*}hea{d_i} = softmax\left( {\frac{{\left( {{Q_i}} \right){{\left( {{K_i}} \right)}^ \top }}}{{\sqrt D }}} \right){V_i},\end{equation*}$$

where

$$\begin{equation*}{\rm{\ }}{Q_i} = \ XW_i^Q,\ \ {K_i} = \ XW_i^K,\ \ {V_i} = \ XW_i^V.\end{equation*}$$

The self-attention mechanism is described as mapping a query and a set of key-value pairs to an output sequence, where the query, key, and value are all matrices: query ${Q_i} = \ [ {q_1^i, \ldots ,q_n^i} ]$, key ${K_i} = \ [ {k_1^i, \ldots ,k_n^i} ]$ and value ${V_i} = \ [ {v_1^i, \ldots ,v_n^i} ]$. These matrices are the inner products of $X$ and the weight matrices $W_i^Q,\ W_i^K,$ and $W_i^V$ of size $D \times D$ that are learned, where $D$ is the input and output vector dimension ($D$= 120 in this study). In the scaled dot-product attention mechanism, each $head$ calculates the next hidden state by computing the attention-weighted sum of the value vector $v$. An attention coefficient is the output of the softmax function applied to the dot product of the query and key $( {{Q_i}} ){( {{K_i}} )^ \top }$ divided by $\sqrt D$. Finally, the $H$$head$ results calculated by different sets of $\{ {W_i^Q,\ W_i^K,\ W_i^V} \}$ are concatenated, and the inner product between this concatenated matrix and ${W^O}$ yields the output sequence$\ C$. After the transformer layer process including multi-head attention is performed six times, the informative base embedding denoted $Z$ is obtained (see the Supplementary Data and Supplementary Figure S1 for more detailed explanation about the self-attention mechanism.)

### Masked language modelling (MLM)

MLM is a task that masks a part of the input RNA sequence and predicts the masked part using the surrounding bases. The MLM task performs a base embedding so that the masked part can be restored, which enables context-sensitive embedding. First, 15% of the bases are randomly selected in a given RNA sequence for training. Next, one of the following three actions is performed on the selected base in the input: 80% of the selected bases are replaced with a token indicating an unspecified base (denoted [mask] in Figure 2), 10% are randomly substituted with one of the other three bases, and the remaining 10% of the selected bases are unchanged from their original base. The MLM task trains the model to maximize the probability of correctly predicting the selected 15% of the RNA bases at the output. In this training model, a classification layer is built on top of the output of the transformer layer. Finally, the output probability of each base is calculated using the softmax function. The cross-entropy function is used as the loss function. The pre-training set for the MLM task consists of 762 370 sequences generated from 76 237 human ncRNA sequences obtained from RNAcentral (21) by taking 10 copies of each ncRNA and applying 10 different mask patterns to each.

### Structural alignment learning (SAL)

The SAL task, which performs a base embedding task to learn the relationship between two RNA sequences, is based on RNA structural alignment. RNA structural alignment aligns multiple RNA sequences by inserting gaps between bases so that the conserved secondary structures are aligned in the same column. The SAL task aims to obtain closer embeddings for bases in the same column of reference alignment and obtain secondary structure embeddings by training based on the RNA structural alignment. The Rfam seed alignment for each family is downloaded from Rfam (19) as the reference structural alignment for the SAL task. To define the loss function in the SAL task, we introduce the $\Omega$ matrix, which is defined for a pairwise alignment of two RNA sequences and is intended to be used as a score matrix when calculating the pairwise alignment. Let $Z\ = \ [ {{z_1}, \ldots ,{z_n}} ]$ and $Z^{\prime}\ = \ [ {z_1^{\prime}, \ldots ,z_m^{\prime}} ]$ denote the embedded representations output from the transformer layer for the input of two RNA sequences of length *n* and *m*. Each element ${\omega _{ij}}$ in the $\Omega$ matrix is defined to be the normalized inner product between ${z_i}$ and $z_j^{\prime}$:

$$\begin{equation*}{\rm{\ }}{\omega _{ij}} = \frac{{{z_i} \cdot z_j^{\rm{^{\prime}}}}}{{\|{z_i}\|\|z_j^{\rm{^{\prime}}}\|}}{\rm{\ }}.\end{equation*}$$

The loss function in the SAL task is defined to increase ${\omega _{ij}}$ at the matched position in the reference alignment so that a sequence alignment algorithm such as the Needleman–Wunsch algorithm produces the reference alignment.

A simple way to implement this loss function in the SAL task is to apply binary classification learning with respect to ${\omega _{ij}}$. That is, ${\omega _{ij}}$ in the aligned position is trained to 1, and ${\omega _{ij}}$ in an unaligned position is trained to 0. However, this causes strong overfitting. To alleviate this problem, we apply a machine learning method called a structured support vector machine (22,23) to the pre-training phase in the SAL task. Let the alignment between a pair of RNA sequences $x\ = {x_1}\ , \ldots ,{x_n}$ and $x^{\prime} = x{^{\prime}_1}\ , \ldots ,x{^{\prime}_m}$ be represented by a series of matched (aligned) positions ($i,j$) and gap insertion positions ($i, -$) or ($ - ,j$), where $1\ \le i \le n,1 \le j \le m$. For a given training dataset $D$ consisting of triplets ($x,x^{\prime},y$), where $x$ and $x^{\prime}$ are a pair of RNA sequences and $y$ is the corresponding reference alignment between $x$ and $x^{\prime}$, we aim to find a set of parameters *w* that minimize the following loss function $L$:

$$\begin{equation*}L = \mathop \sum \limits_{\left( {x,x^{\prime},\ y} \right) \in D} \left\{ {f\left( {x,x^{\prime},\hat{y}} \right) + \ \Delta \left( {y,\ \hat{y}} \right) - f\left( {x,x^{\prime},y} \right) + \ \lambda \|w{\|_2}\ } \right\},\end{equation*}$$

where $f$ is the function that returns the alignment score $y$ between $x$ and $x^{\prime}$. The term $\lambda \|w{\|_2}$ in the above formula is the L2 regularization term to avoid overfitting, where $w$ refers to the parameters of the entire model, $\parallel w{\parallel _2}$ is the squared value of the model parameters and $\lambda$ is a parameter that controls the strength of regularization. The alignment score is calculated as the sum of the ${\omega _{ij}}$ value at the matched position ($i,j$) and the gap score at the gap insertion positions ($i, -$) or ($ - ,j$). $\hat{y}$ is the predicted alignment path calculated by the Needleman–Wunsch algorithm to maximize the sum of the alignment score $f( {x,x^{\prime},\hat{y}} )$and the margin term $\Delta ( {y,\ \hat{y}} )$. The margin term $\Delta ( {y,\ \hat{y}} )$ defines the difference between the reference alignment and the predicted alignment as follows:

$$\begin{eqnarray*} \Delta \left( {y,\ \hat{y}} \right) &=& \ {\delta ^{FN}} \times \left( {{\rm{the\ number\ of\ positions\ included\ in\ }}y\ {\rm{but\ not\ in}}\ \hat{y}} \right)\nonumber\\ &&+ {\delta ^{FP}} \times \left( {{\rm{the\ number\ of\ positions\ included\ in\ }}\hat{y}\ {\rm{but\ not\ in}}\ y} \right). \end{eqnarray*}$$

Here, ${\delta ^{FN}}$ and ${\delta ^{FP}}$ are hyperparameters that control the trade-off between sensitivity and specificity for learning parameters. By default, we used ${\delta ^{FN}}$ = 0.05 and ${\delta ^{FP}}$ = 0.1, which were determined by the grid-search optimization in the range 0.01–0.30. Decreasing the loss function $L$ brings the predicted alignment closer to the reference alignment.

### RNA structural alignment

A pairwise RNA sequence alignment based on the base embedding is calculated using the Needleman–Wunsch algorithm using the $\Omega$ matrix as the score matrix, which is derived from the training of SAL and MLM tasks. The match score in position $( {i,\ j} )$ is ${\omega _{ij}}$ in the score matrix $\Omega$, and the gap opening score and gap extension score are set to -1 and -0.1, respectively. As the MLM task enables the position- and context-sensitive embedding and SAL task enables the structural information embedding, the Needleman–Wunsch algorithm, a simple sequence alignment algorithm, is expected to generate RNA structure alignments using the $\Omega$ matrix derived from the SAL and MLM tasks. Note that the time complexity of the Needleman–Wunsch algorithm is *O*(*n*^2^) for the input RNA sequence of length *n*.

### RNA family clustering

RNA family clustering is performed as the second evaluation test to confirm the quality of the informative base embedding. A similarity measure between two RNA sequences with respect to soft symmetric alignment (24) is defined as follows. Let $Z\ = \ [ {{z_1}, \ldots ,{z_n}} ]$ and $Z^{\prime}\ = \ [ {z_1^{\prime}, \ldots ,z_m^{\prime}} ]$ denote the embedded representations output from the transformer layer for the input of a pair of RNA sequences of length *n* and *m*. The similarity $\hat{s}$ between the two RNA sequences is defined to be the weighted sum of the normalized inner product between all ${z_i}$ and $z_j^{\prime}$ pairs:

$$\begin{equation*}\hat{s}\ = \frac{1}{A}\ \mathop \sum \limits_{i\ = \ 1}^n \mathop \sum \limits_{j\ = \ 1}^m {a_{ij}}{\omega _{ij}},\ {\omega _{ij}}\ = \frac{{{z_i} \cdot z_j^{\prime}}}{{\|{z_i}\|\|z_j^{\prime}\|}}\ ,\ A\ = \mathop \sum \limits_{i = 1}^n \mathop \sum \limits_{j = 1}^m {a_{ij}}\ ,\end{equation*}$$

where ${a_{ij}}$ is

$$\begin{equation*}{\rm{\ }}{a_{ij}} = {\alpha _{ij}}\ + {\beta _{ij}} - {\alpha _{ij}}{\beta _{ij}},\end{equation*}$$

$$\begin{equation*}{\rm{\ }}{\alpha _{ij}} = \frac{{exp\left( {{\omega _{ij}}} \right)}}{{\mathop \sum \nolimits_{k = 1}^m exp\left( {{\omega _{ik}}} \right)}}\ ,\end{equation*}$$

$$\begin{equation*}{\rm{\ }}{\beta _{ij}} = \frac{{exp\left( {{\omega _{ij}}} \right)}}{{\mathop \sum \nolimits_{k = 1}^n exp\left( {{\omega _{kj}}} \right)}}{\rm{\ }}.\end{equation*}$$

The similarity $\hat{s}$ is calculated for all pairs of ncRNA sequences to be clustered, and a classification matrix of size $N \times N$ is created, where $N$ is the number of RNA sequences in the test dataset. We applied spectral clustering to the rows of the classification matrix by considering each row of the $N$-dimensional vector a cluster indicator. To confirm the improvement in the embedding quality by the SAL task, we compared the clustering accuracy when using only the MLM task with that when using the two tasks together.

### Existing methods for RNA structural alignment

There is a family of Sankoff-style algorithms for structural pairwise alignment that simultaneously predicts the optimal alignment and the consensus secondary structure. For example, Dynalign and Foldalign (13,25) use thermodynamic models to find the minimum free energy consensus structures, while PARTS (26) uses a probabilistic model based on the pseudo-energy obtained from base-pairing probabilities and alignment probabilities to find the most likely structural alignment. While Sankoff-style algorithms yield a high alignment accuracy, the naive implementation is computationally expensive, with a time complexity of *O*(*n*^6^) for RNA sequences of length *n*. PMcomp takes base-pairing probability matrices generated using McCaskill’s algorithm as the input and incorporates the energy information of each sequence into these matrices to quickly find common secondary structures and alignments (27). Although LocARNA (12) is based on the PMcomp model, a time complexity of *O*(*n*^4^) is achieved by simplifying the dynamic programming method utilizing the fact that the base-pairing probability matrix is actually sparse. SPARSE (28) takes further advantage of this sparsity based on the conditional probabilities of bases and base pairs in the loop region of the RNA secondary structure, achieving a quadratic improvement in the computational time over LocARNA. RAF (29) achieves the same time complexity as SPARSE by utilizing the sparseness of alignment candidates. DAFS is a state-of-the-art accurate structural alignment program utilizing integer programming technique (30) and its time complexity is *O*(*n*^3^). R-Coffee is a multiple RNA alignment package that takes a similar strategy with our study by utilizing an alignment-scoring scheme that incorporates secondary structure information (31) and its time complexity is *O*(*n*^2^). As R-Coffee makes use of the base-pairing probability calculated with McCaskill’s algorithm, it is considered as a type of structural alignment algorithm. TOPAS is a network-based scheme for pairwise structural alignment of RNAs that can handle pseudoknots (32), and its time complexity is *O*(*n*^4^) in the worst case. TOPAS employs graph data structures to represent the RNA secondary structure including pseudoknots and designs an efficient algorithm to calculate an alignment of two graph structures by matching two nodes in two different graphs. Finally, MAFFT v7 (33), which uses Kimura’s two-parameter model (34) as the score matrix, was adopted as the baseline for RNA sequence alignment. Note that MAFFT is a sequence-based alignment algorithm that does not take RNA structure information into account. The list of command, options, package and link information for existing alignment methods is provided in the Supplementary Table S1.

### Existing methods for RNA family clustering

The clustering accuracies of the state-of-the-art methods GraphClust (15), EnsembleClust (16) and CNNclust (18) were compared. CNNclust is a deep learning-based algorithm that performs supervised learning in which the RNA family class is given as a label. CNNclust can classify RNA families that are not used for training by calculating the similarity score matrix for all pairs of input sequences. We performed experiments with CNNclust using different RNA family groups between training and testing. In contrast, GraphClust is an unsupervised learning algorithm that does not require the RNA family class to be a label and achieves alignment-free clustering with some exceptions. GraphClust employs a graph kernel approach to obtain feature vectors that contain both sequence and secondary structure information. These vectors representing RNA sequences are clustered with a linear time complexity over the number of sequences using a hashing technique. Finally, EnsembleClust calculates the similarity between two ncRNAs using the expected structural alignment and then applies hierarchical clustering based on the similarity. The list of command, options, package and link information for existing clustering methods is provided in the Supplementary Table S1.

### Sequence motif detection using a self-attention mechanism

We extracted the sequence motifs specific to each RNA family by focusing on the self-attention mechanism, which determines where to focus on the input embedding vectors $X\ = \ [ {{x_1}, \ldots ,{x_n}} ]$ of the input RNA sequence $r\ = {r_1}\ , \ldots ,{r_n}$ when generating the output sequence. The attention coefficient sequence $M\ = \ [ {{m_1}, \ldots ,{m_n}} ]$, called attention map, that is calculated for the input sequence $r\ = {r_1}\ , \ldots ,{r_n}$ is defined as follows:

$$\begin{equation*}M{\rm{\ }} = \mathop \sum \limits_{h = 1}^H \mathop \sum \limits_{i = 1}^n softmax\left( {\frac{{\left( {q_i^h} \right){{\left( {{K_h}} \right)}^ \top }}}{{\sqrt D }}} \right){\rm{\ }}.\end{equation*}$$

The base ${r_i}$ at position $i$ with a high ${m_i}$ value is identified as part of the motif. Thus, the attention map helps discover the sequence motif since it indicates a base that is important for training tasks (see the Supplementary Data and Supplementary Figure S2 for more detailed explanation about RNA motif detection using self-attention map).

### Measures of the accuracies of alignment and clustering

Structural alignment accuracy was measured using sensitivity, positive predictive value (PPV) and F1 score, which are calculated as follows. The number of true positives (TP) (or false positives [FP]) is the number of positions $( {i,\ j} )$ in the predicted alignment that belong (or do not belong) to the reference alignment. The sensitivity of the predicted alignment is TP divided by the number of positions in the reference alignment, and the PPV is TP divided by the number of positions in the predicted alignment. The F1 score is the harmonic mean of sensitivity and PPV.

Clustering accuracy was measured with the Rfam family as the true reference class. Three indices, namely, the adjusted Rand index (ARI), homogeneity, and completeness, were used to evaluate the clustering performance. The ARI is a measure of how well two types of clustering results match. ARI takes a real number from -1 to 1: if the value of ARI is -1, the two clustering results do not match at all, while a value of 1 indicates that they completely match. In this study, the ARI reflects how close the predicted clustering result is to the true reference class composed of the Rfam family.

The ARI is derived from the Rand index (RI), defined as follows:

$$\begin{equation*}RI\ = \ \frac{{TP + TN}}{{TP + TN + FP + FN}}\end{equation*}$$

$$\begin{equation*}E\ = \ \frac{{\left( {TP + FP} \right)\left( {TP + FN} \right) + \left( {TN + FP} \right)\left( {TN + FN} \right)}}{{TP + TN + FP + FN}}\end{equation*}$$

$$\begin{equation*}ARI\ = \ \frac{{\left( {TP + TN} \right) - E}}{{\left( {TP + TN + FP + FN} \right) - E}}\end{equation*}$$

where TP is the number of RNA sequences of the same Rfam family in the same predicted cluster, TN is the number of RNA sequences of a different Rfam family in different predicted clusters, FP is the number of RNA sequences of different Rfam families in the same predicted cluster, and FN is the number of RNA sequences of the same Rfam family in different predicted clusters. Homogeneity is a measure of the proportion of RNA sequences of a single Rfam family that belong to a single predicted cluster, and completeness measures the proportion of RNA sequences of a particular Rfam family that are assigned to the same predicted cluster.

### Datasets

For the pre-training of the MLM task, 76 237 human-derived small ncRNAs with lengths ranging from 20 to 440 bases from RNAcentral (21) were utilized.

In the training of the SAL task, two types of datasets, named TrainSet-A and TrainSet-B, were devised. In both datasets, the pairwise structural alignment extracted from Rfam alignment (19) was used. TrainSet-A consists of RNA sequences sampled from seed RNA sequences in 36 RNA families in which all families were overlapped with the following structural alignment benchmark dataset. TrainSet-B consists of RNA sequences from all RNA families (3983 families) in Rfam database except the RNA families used in the benchmark dataset BRAliBase2.1 k2 database (35). In other words, the training and test datasets do not overlap with respect to the RNA family.

For the structural alignment benchmark, we utilized the BRAliBase2.1 k2 database (35) used in the previous study as the gold standard benchmark dataset. Sequence pairs containing unknown bases were eliminated. A total of 8587 RNA sequence pairs with an average length of approximately 100 bases were used for the benchmark test dataset. The lists of RNA families in BRAliBase2.1 k2 database and TrainSet-A of the SAL task are provided in the Supplementary Data. Note that no alignment overlapped between TrainSet-A and the benchmark test dataset.

To evaluate the clustering accuracy of RNABERT, the test dataset was collected from the BRAliBase2.1 database. The multiple alignment of each ncRNA family provided by the database was treated as a true reference cluster, and each ncRNA sequence in the multiple alignment was treated as a member sequence. All reference clusters with a sequence identity of <40% were selected. The dataset contained 37 RNA sequences and 12 RNA families. The RNA sequences used in the RNA family clustering test did not overlap with those used for the pre-training of the SAL task.

### Implementation

The RNABERT model was implemented using PyTorch for deep learning. All experiments were run on Linux Red Hat 4.8.5–2 (GPU: Tesla v100, CPU: Intel(R) Xeon(R) Gold 6148). Optuna (36) was used to find the optimal hyperparameters for the MLM task. The hyperparameters optimized for the transformer layer were the number of attention heads, number of transformer layers, feature size, activation function, and training algorithms, including Adam, AdaGrad and momentum stochastic gradient descent (SGD). In the MLM task, 5-fold cross-validation was performed, and the hyperparameters were determined to maximize accuracy.

## RESULTS

### Pre-training of base embedding encodes properties of RNA secondary structure

**Figure 3.**
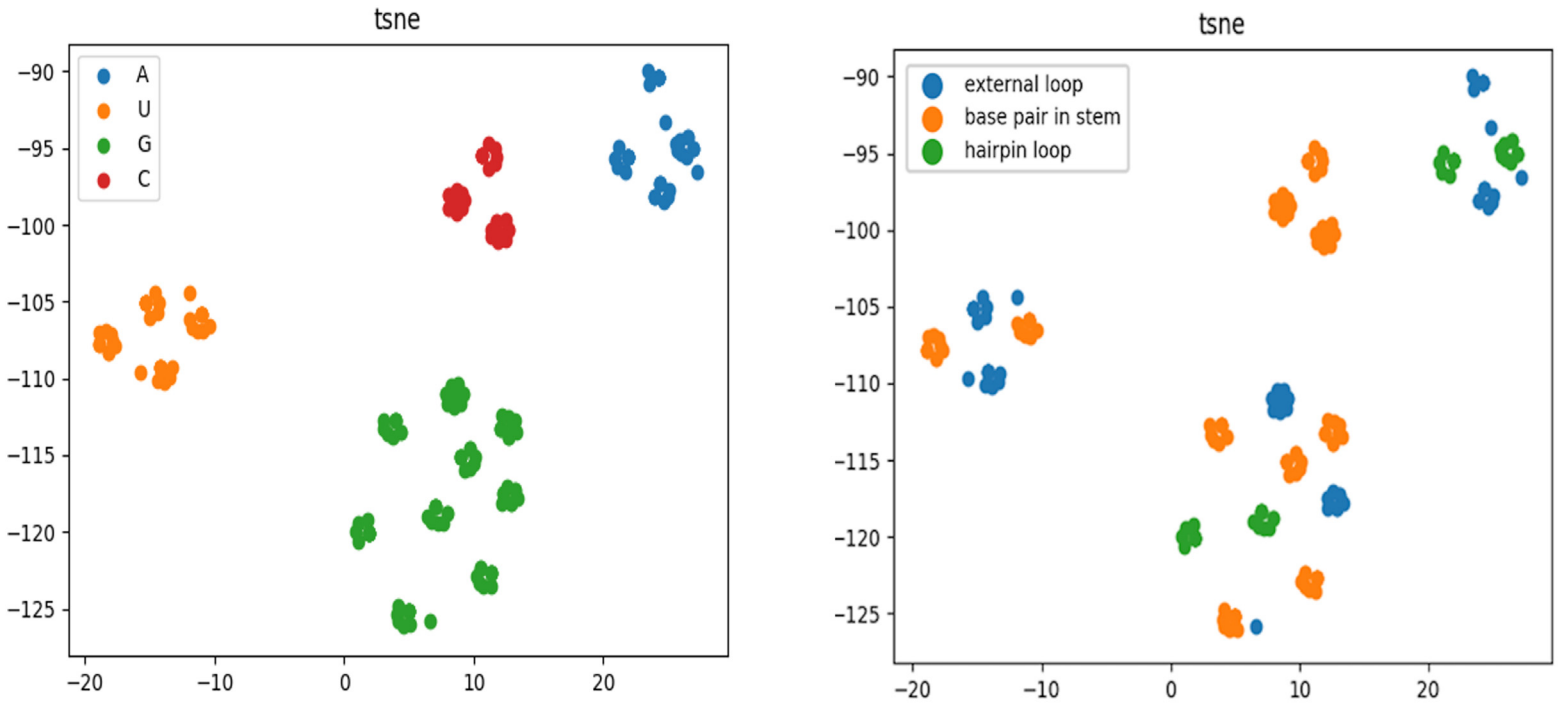
Visualization of RNA base embedding. Shown is a t-SNE projection from a 120-dimensional embedded space to a two-dimensional space. RNA base embeddings are visualized with colours according to the type of RNA base (left) and the type of characteristic secondary substructure (right). The embedding space adequately represents the clusters for four RNA bases (left) and the subclusters for characteristic secondary structures (right). The secondary structure of an RNA is basically composed of a combination of six substructures; hairpin loop, base pair in stem, bulge and internal loop, multibranch loop, external loop at 3′, and external loop at 5′. The Supplementary Figure S4 illustrates the six substructures.

To investigate whether RNABERT acquired an informative base embedding to encode four RNA bases and secondary structure information, the embedded representations output from the transformer layer for a set of RNA sequences were projected into two-dimensional space using *t*-distributed stochastic neighbour embedding (t-SNE) (37), which is a dimension reduction algorithm for mapping high-dimensional data to low dimensions. Figure 3 shows the result of mapping the 120-dimensional vector of each base into a two-dimensional space (with the option ‘n_components = 2’). In the dimension reduction by t-SNE, the distance relationship between bases embedded in the original 120-dimensional space is projected in two dimensions so as to be preserved as much as possible. The embedding space adequately represents the clusters for four RNA bases (Figure 3, left) and the subclusters for characteristic secondary substructures (Figure 3, right). Figure 3 shows that the RNA base embedding is globally separated by four RNA bases and locally separated by characteristic secondary substructures (hairpin loop, base pair in stem and external loop) within each RNA base. This result clearly shows that RNABERT embedding using pre-training with SAL and MLM tasks succeeded in encoding not only base (nucleotide) information but also secondary structure information (see the Figure S3 for t-SNE projection of embedding for all secondary substructures. The secondary structure of an RNA is basically composed of a combination of six substructures; hairpin loop, base pair in stem, bulge and internal loop, multibranch loop, external loop at 3′ and external loop at 5′. The Supplementary Figure S4 illustrates the six substructures.

### RNA structural alignment result

Table 1 summarizes the performance evaluation results based on the BRAliBase2.1 k2 database for our RNA structural alignment method, RNABERT trained on TrainSet-A and TrainSet-B, and for the state-of-the-art algorithms for RNA sequence alignment. As shown in Table 1, RNABERT trained on TrainSet-A outperformed the existing state-of-the-art structural alignment algorithms in all three measures of accuracy. On the other hand, the performance of RNABERT trained on TrainSet-B was still sufficiently high, similar to other structural alignment algorithms. This result indicates that RNABERT has sufficient generalization ability when trained on a large set of RNA families.

**Table 1.** RNA structural alignment accuracies and computational times (shown in seconds) of RNABERT and state-of-the-art algorithms

	Sensitivity	PPV	F1	Time (s)
RNABERT (TrainSet-A)	0.881	0.947	0.913	288
RNABERT (TrainSet-B)	0.851	0.932	0.890	284
LocaRNA	0.862	0.922	0.891	13,221
SPARSE	0.848	0.931	0.888	4,216
RAF	0.865	0.938	0.900	1,423
PARTS	0.860	0.931	0.894	432,585
Dynalign2	0.706	0.913	0.796	601,104
R-Coffee	0.842	0.934	0.886	878
TOPAS	0.879	0.938	0.908	2,103
Foldalign	0.861	0.922	0.890	451,112
DAFS	0.862	0.936	0.897	2,210
MAFFT	0.810	0.901	0.853	1,282

In terms of computation time, RNABERT was faster than the existing state-of-the-art algorithms and even faster than the sequence-based (non-structural) alignment algorithm MAFFT. The alignment computation of RNABERT consists of three sub-procedures: the first procedure (transformer) obtains the embedding of each base, the second procedure calculates the match score between the two input sequences, and the third procedure calculates the alignment by the Needleman–Wunsch algorithm. The first two procedures can be accelerated by GPU computation, and the Needleman–Wunsch algorithm is a simple algorithm that requires a computation time of *O*(*n*^2^) for two sequences of length *n*. We achieved high-speed computation by implementing the deep learning algorithm using Python and PyTorch while implementing the Needleman–Wunsch algorithm in C++. Note that the loading time of the transformer model into the GPU was excluded from the time measurement of pairwise alignment by RNABERT. The typical amount of time needed to load the transformer model onto GPU was around 4.376 s. In addition, the maximum memory consumption for the RNA structural alignment was around 35.2G bytes in RNABERT.

**Figure 4.**
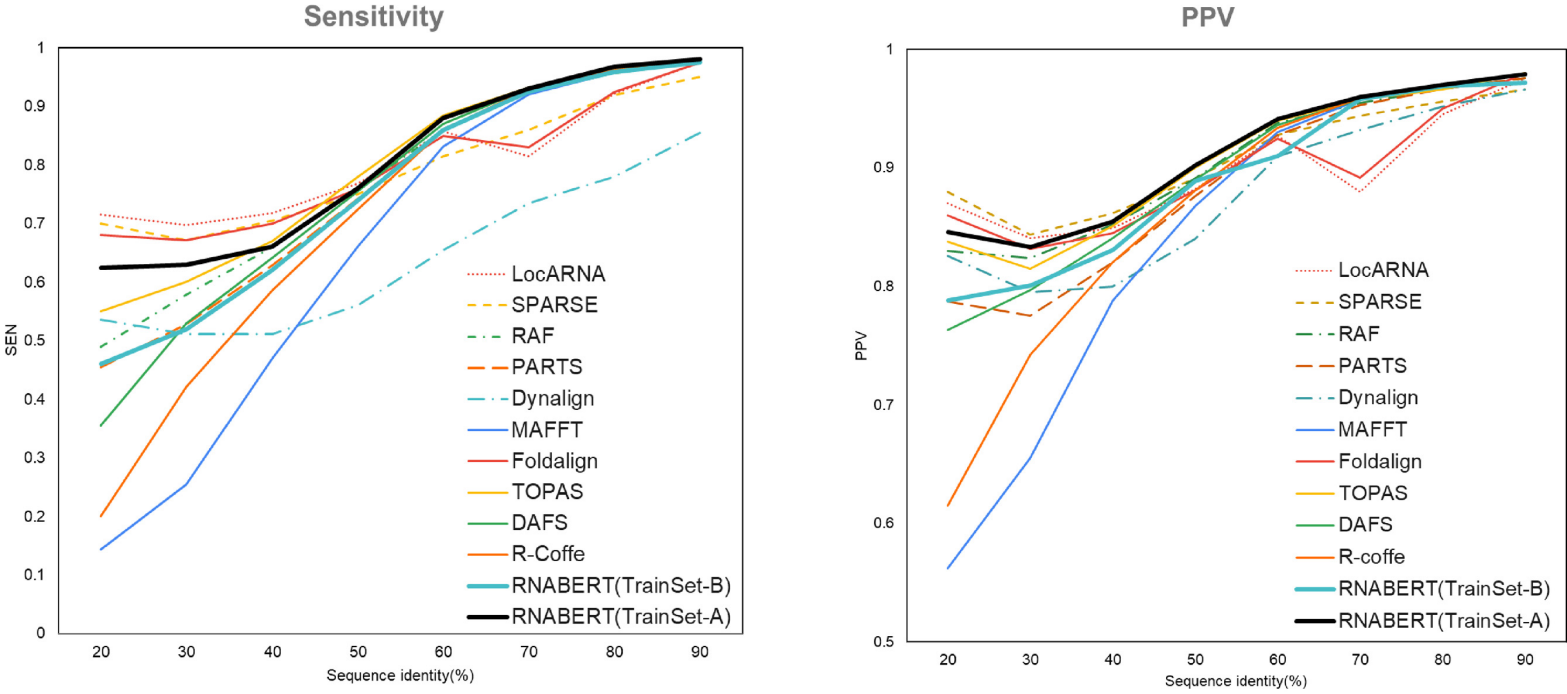
SEN and PPV score plots for pairwise RNA structural alignments using RNABERT(TrainSet-A), RNABERT(TrainSet-B), LocARNA, SPARSE, RAF, PARTS, Dynalign, Foldalign, R-Coffee, TOPAS, DAFS and a sequence-based alignment using MAFFT.

Figure 4 shows the sensitivity (denoted SEN) and PPV curves calculated for each RNA sequence alignment algorithm. These values were plotted by sequence identity. As shown in Figure 4, RNABERT yielded very accurate structural alignment results and outperformed the existing state-of-the-art structural alignment algorithms where the sequence identity exceeded 50%. At lower sequence identities, the alignment accuracy of RNABERT(TrainSet-A) was slightly lower than those of LocARNA, SPARSE and Foldalign, which required larger computation times, and was higher than that of RAF, which exhibited the fastest computational time among the existing structural alignment algorithms. All existing Sankoff-style algorithms conduct RNA secondary structure predictions to calculate the distances and similarities between RNA sequences. On the other hand, RNABERT does not explicitly use secondary structure predictions, which implies that the RNA base embedding efficiently captures structural information. In particular, for sequences with very low sequence identities, the accuracy of the sequence-based alignment MAFFT tends to decrease, while RNABERT and the existing structural alignment algorithms maintain high accuracy.

### RNA family clustering results

Table 2 shows the ARI, homogeneity and completeness of our RNA clustering method, RNABERT, and those of the state-of-the-art tools for RNA family clustering. RNABERT(TrainSet-A) with the MLM and SAL tasks achieved the highest ARI and completeness among all state-of-the-art tools. The existing methods all utilize RNA secondary structure predictions to calculate the distances and similarities between RNA sequences. This implies that the RNABERT base embedding, which does not explicitly use secondary structure prediction but uses the same RNA family for SAL task, efficiently captures structural information. On the other hand, the performance of RNABERT(TrainSet-B) trained on different RNA families is less accurate compared with GraphClust and similar with CNNclust. This result indicates that the SAL task designed for effective structural alignment, but not for family clustering, is not sufficient for unknown RNA family clustering.

**Table 2.** RNA family clustering accuracy. The ARI, homogeneity and completeness are shown for RNABERT and the state-of-the-art tools for RNA family clustering

	ARI	Homogeneity	Completeness	Time (s)
RNABERT (TrainSet-A) (MLM + SAL)	0.268	0.663	0.758	28.69
RNABERT (TrainSet-B) (MLM + SAL)	0.187	0.568	0.664	27.16
RNABERT (MLM)	0.177	0.556	0.663	27.81
CNNclust	0.189	0.612	0.642	17.45
EnsembleClust	0.200	0.587	0.661	11.32
GraphClust	0.243	0.746	0.666	520.22

### RNA motif

**Figure 5.**
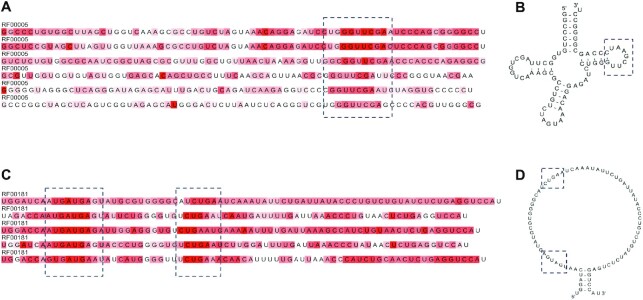
Extracted sequence motifs of tRNA (**A**), (**B**) and snoRNA families (**C** and **D**). (A) and (C) are visualizations of the attention map at each base. Bases with a darker backgrounds have higher attention map values.

Several well-known sequence motifs in the snoRNA and tRNA families were identified by observing the attention maps. Attention maps, which indicate the ratios of contribution to the MLM task, were extracted from the final transformer layer of RNABERT, and sequence motifs were detected from the attention maps. The ‘UUCGA’ sequence motif shown in Figure 5A is typical in the T loop of tRNA (38). This motif is specifically present in TRT-AGT6-1 (tRNA gene with anticodon AGT), as displayed in the secondary structure in Figure 5B. The motifs depicted in Figure 5C are the typical motifs ‘UGAUGA’ and ‘CUGA’ present in the snoRNA C/D box (39,40). These motifs are specifically present at SNORD113-7, as displayed in the secondary structure in Figure 5D.

## DISCUSSION

In this study, we performed two tasks to obtain informative base embeddings. The MLM task is a fundamental step in the original BERT algorithm, whereas SAL is a novel RNA sequence-specific task introduced in this study. To determine whether these tasks effectively incorporate RNA secondary structure information into base embeddings, we performed two tests, RNA clustering and sequence alignment.

Sankoff-style algorithm provides high structural alignment accuracy, but these algorithms are usually very complex in both time and space. Unlike the many structural alignment algorithms based on the Sankoff algorithm, RNABERT does not explicitly consider RNA folding and boasts a high structural alignment accuracy. This is considered to be evidence that the base embedding encodes the secondary structure information specific to RNAs. Furthermore, while RNABERT achieves the same accuracy as Sankoff-style algorithms, it is much faster because it uses a simple sequence-based alignment algorithm. In fact, the time complexity of the RNABERT algorithm is only *O*(*n*^2^) for two sequences of length *n*.

SPARSE (28) achieves a quadratic improvement in the computational time of Sankoff-style algorithms for simultaneous alignment and folding by assuming that RNA secondary structures are sparse. On the other hand, RNABERT similarly achieves a quadratic computational time improvement by reducing the RNA structural alignment problem to a sequence alignment problem based on the pre-training of base embeddings. In this way, the computational time of RNABERT was an order of magnitude faster than that of SPARSE, as revealed in this study.

Performance evaluation was done for two types of training datasets, TrainSet-A and TrainSet-B. TrainSet-A contains the same RNA families as the benchmark test dataset while TrainSet-B has no RNA family overlap with the test dataset. When TrainSet-A was used, RNABERT exhibited a superior accuracy than state-of-the-art existing structural alignment methods. When TrainSet-B was used, the performance of RNABERT was still sufficiently high and comparable to the one using TrainSet-A. This result shows that RNABERT has succeeded in proposing a new scoring scheme for sequence-based alignment algorithms to accomplish RNA structural alignment and has sufficient generalization ability. It has to be noted that with the development of high-throughput sequencing, hundreds of thousands of ncRNAs have been detected, but many have not been annotated yet. In fact, 86% (24 972 896) of the 28 895 596 ncRNAs present in RNAcentral do not have gene ontology (GO) annotations. Therefore, fast and accurate structural alignment of unknown sequences of existing RNA families is still practically valuable and RNABERT could contribute to the annotation of such novel transcripts.

The base embeddings obtained by RNABERT are applicable to various fields in RNA informatics. One immediate problem is the multiple structural alignment of RNA sequences. RNABERT can be expected to accomplish this task by combining existing sequence-based multiple alignment algorithms such as MUSCLE (41) and MAFFT (33) with the score matrix $\Omega$ and informative base embedding. Another area most likely to improve with the application of RNABERT is the prediction of RNA secondary structures. Since the base embeddings contain information on secondary structures, RNABERT is expected to contribute to the prediction of RNA secondary structures (33,41,42). Similarly, base embeddings can be applied to the RNA interactome (RNA–protein interaction, RNA–RNA interaction), in which the RNA secondary structure acts on the interaction between molecules. In order to accomplish such secondary structure-related problems, it would be a better approach to incorporate the secondary structure prediction as another pre-training task in the pre-training process of RNABERT. Finally, while this study has not addressed RNA modification (e.g. m6A, m1A), these findings may be helpful for utilizing this information for the more precise modelling of base embeddings.

## DATA AVAILABILITY

The codes, pre-trained RNABERT model, and all datasets used in this study are available at https://github.com/mana438/RNABERT.git.

## Supplementary Material

lqac012_Supplemental_File

## Notes

Present address: Manato Akiyama, Biosciences and Informatics, Keio University, Yokohama, 223-8522, Japan.
