# Supplementary material for: Informative RNA base embedding for RNA structural alignment and clustering by deep representation learning
Source: NAR Genom Bioinform. 2022 Feb 22;4(1):lqac012. doi: 10.1093/nargab/lqac012 (PMC8862729; doi:10.1093/nargab/lqac012)
Supplement: lqac012_Supplemental_File [file lqac012_supplemental_file.pdf]

Supplementary Information:

“Informative RNA base embedding for RNA structural alignment and clustering by deep representation learning”

Manato Akiyama and Yasubumi Sakakibara*

Supplemental Sections

Self-Attention Mechanism

Figure S1 illustrates the single-head case of the self-attention mechanism. The transformer is an encoder-decoder type of feed-forward neural network. The self-attention function for an encoder-decoder neural network is a dot-product attention formulated as

$$attention = softmax(Target \cdot Source^{\top}) \cdot Source ,$$

where $Souce$ represents the encoder layer and $Target$ represents the decoder layer. The BERT algorithm generalized it by considering $Target$ as (search) query Q and separating $Source$ into key K and value V, formulated as:

$$attention = softmax(Q \cdot K^{\top}) \cdot V .$$

In this formulation, the attention function computes an output (attention weight) based on a query (Q) and a set of key-value pairs (K, V). The key-value pairs (K, V) can be considered as a kind of dictionary. By separating $Source$ into key K and value V, the dot-product between query Q and key K plays a role to measure the relevance of the value V for query Q (how much it has an attention). These Q, K and V are calculated by linear projection from the input X with learnable parameters W^Q, W^K and W^V, formulated as:

$$Q = XW^Q, K = XW^K, V = XW^V.$$

RNA Motif Detection using Self-Attention Map

The attention map calculates the inner product between the query vector of each base and the key vector of the other bases in the input RNA sequence, then measures the relevance of the base with the other bases, as illustrated in the supplemental Figure S1. The supplemental Figure S2 shows the strength of the relevance of each base, which is represented by the intensity of red. The sequence below in Figure S2 represents the relevance of the 10th base “G” from the left in the upper sequence, which is surrounded by a blue frame. In the Figure S2, arrows are drawn for bases that are particularly relevant to the base “G”. The sum of the relevance calculated for each base is finally defined as an attention map. Thus, the attention map is an index showing how much each

base contributed to the prediction of the pre-training task. Therefore, in the MLM task, the bases that are important for the prediction of the masked base, and in the SAL task, for the prediction of the structural alignment obtain high values in the attention map. Finally, the bases with high attention values are identified as sequence motif.

Datasets

TrainSet-A: The list of 36 RNA families included in the TrainSet-A is

5.8S rRNA, 5S rRNA, Cobalamin, Entero 5 CRE, Entero CRE, Entero OriR, gcvT, Hammerhead 1, Hammerhead 3, HCV SLIV, HCV SLVII, HepC CRE, Histone3, HIV FE, HIV GSL3, HIV PBS, Intron gpII, IRES HCV, IRES Picorna, K chan RES, Lysine, TAR, Retroviral psi, S box, SECIS, sno 14q I II, SRP bact, SRP euk arch, T-box, THI, tRNA, U1, U2, U6, UnaL2, yybP-ykoY.

Benchmark test dataset BRAliBase2.1:

5.8S rRNA, 5S rRNA, Cobalamin, Entero 5 CRE, Entero CRE, Entero OriR, gcvT, Hammerhead 1, Hammerhead 3, HCV SLIV, HCV SLVII, HepC CRE, Histone3, HIV FE, HIV GSL3, HIV PBS, Intron gpII, IRES HCV, IRES Picorna, K chan RES, Lysine, TAR, Retroviral psi, S box, SECIS, sno 14q I II, SRP bact, SRP euk arch, T-box, THI, tRNA, U1, U2, U6, UnaL2, yybP-ykoY.

Supplemental Tables and Figures

Supplemental Table S1. The list of command, options, package, and link information for each existing method.

Programs	command	Package	URL
LocARNA	locarna fasta_file	LocARNA 1.9.2.1	https://rna.informatik.uni-freiburg.de/LocARNA/Input.jsp
SPARSE	sparse fasta_file	LocARNA 1.9.2.1	http://www.bioinf.uni-freiburg.de/Software/SPARSE/
RAF	raf predict fasta_file	1.0.0	http://contra.stanford.edu/contrafold/raf.html
PARTS	parts configuration_file	RNAstructure version 6.4	http://rna.urmc.rochester.edu/RNAstructure.html
Dynalign	dynalign_ii configuration_file	RNAstructure version 6.4	http://rna.urmc.rochester.edu/RNAstructure.html
MAFFT	mafft –globalpair fasta_file	MAFFT version 7.490	https://mafft.cbrc.jp/alignment/software/
Foldalign	foldalign -global fasta_file	Foldalign version 2.5.3	https://rth.dk/resources/foldalign/
TOPAS	TOPAS[fasta_file, base-pairing and alignment probabilities, Alpha= 0.4, Beta= 0.56]	TOPAS version 1.3	https://github.com/bjyoontamu/TOPAS
DAFS	dafs fasta_file	0.0.3	https://github.com/satoken/dafs
R-Coffe	t_coffee fasta_file -mode rcoffee	T-COFFEE Version_13.45.0	http://www.tcoffee.org/Projects/rcoffee/index.html

The “configuration_file” used in PARTS and Dyalign and MATLAB format file for executing programs in TOPAS are available on https://github.com/mana438/RNABERT.git.

Programs	command	Package	URL
CNNclust	python RNApairClassify.py -d -l numpy_files_for_aligned ncRNA_pair -gl gene_anotation_file -v 0 -p CNN_model	CNNclust	http://www.dna.bio.keio.ac.jp/cnn/
EnsembleClust	cluster fasta_file sequence_names	EnsembleClust-1.0	http://bpla-kernel.dna.bio.keio.ac.jp/clustering/
GraphClust	GraphClust.pl --root run_test_1 –fasta fasta_file --config default_file --verbose	GraphClust 0.7.6(docker image)	http://www.bioinf.uni-freiburg.de/Software/GraphClust/

Supplemental Figure S1. Illustration of the single-head case of the self-attention mechanism.

Figure S1

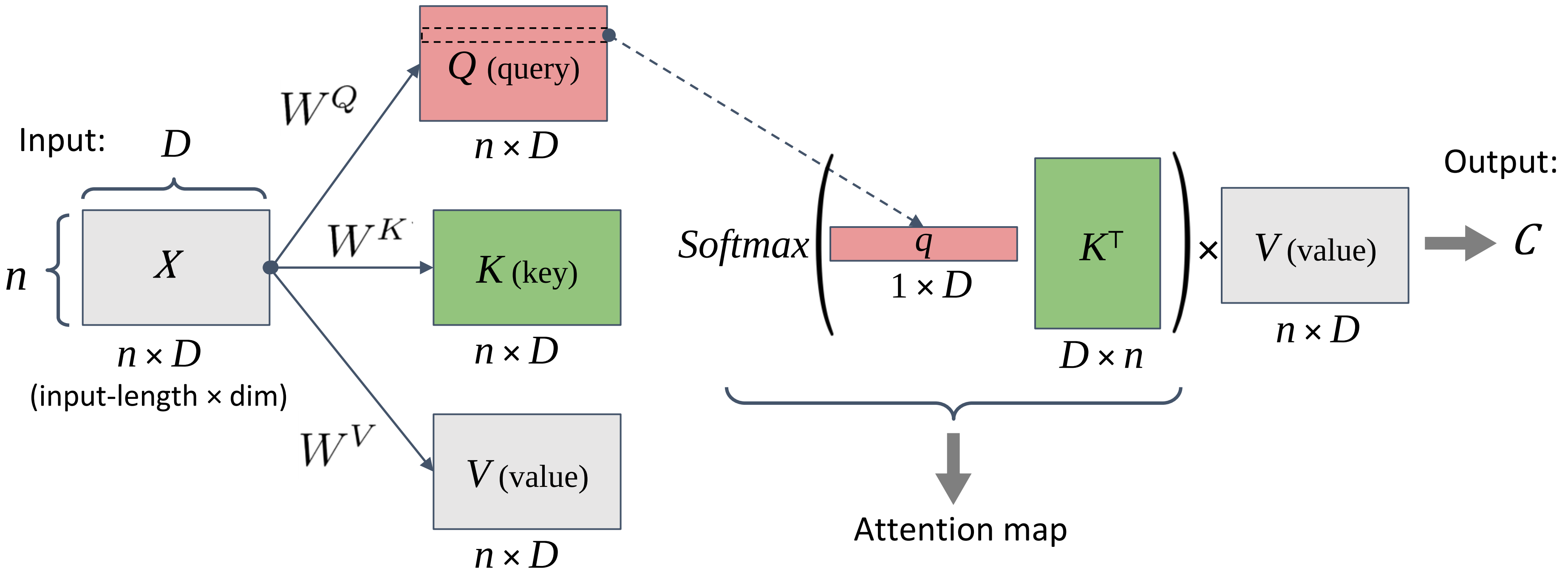

Input:
D
n
X
n × D
(input-length × dim)
W^Q
W^K
W^V
Q (query)
n × D
K (key)
n × D
V (value)
n × D
Softmax
q
1 × D
K^⊤
D × n
×
V (value)
n × D
Output:
C
Attention map

Supplemental Figure S2. Example of RNA motif detection using self-attention map.

The attention map calculates the inner product between the query vector of each base and the key vector of the other bases in the input RNA sequence, then measures the relevance of the base with the other bases, as illustrated in the supplemental Figure S1. The supplemental Figure S2 shows the strength of the relevance of each base, which is represented by the intensity of red. The sequence below in Figure S2 represents the relevance of the 10th base “G” from the left in the upper sequence, which is surrounded by a blue frame. In the Figure S2, arrows are drawn for bases that are particularly relevant to the base “G”. The sum of the relevance calculated for each base is finally defined as an attention map. Thus, the attention map is an index showing how much each base contributed to the prediction of the pre-training task. Therefore, in the MLM task, the bases that are important for the prediction of the masked base, and in the SAL task, for the prediction of the structural alignment obtain high values in the attention map. Finally, the bases with high attention values are identified as sequence motif.

Figure S2

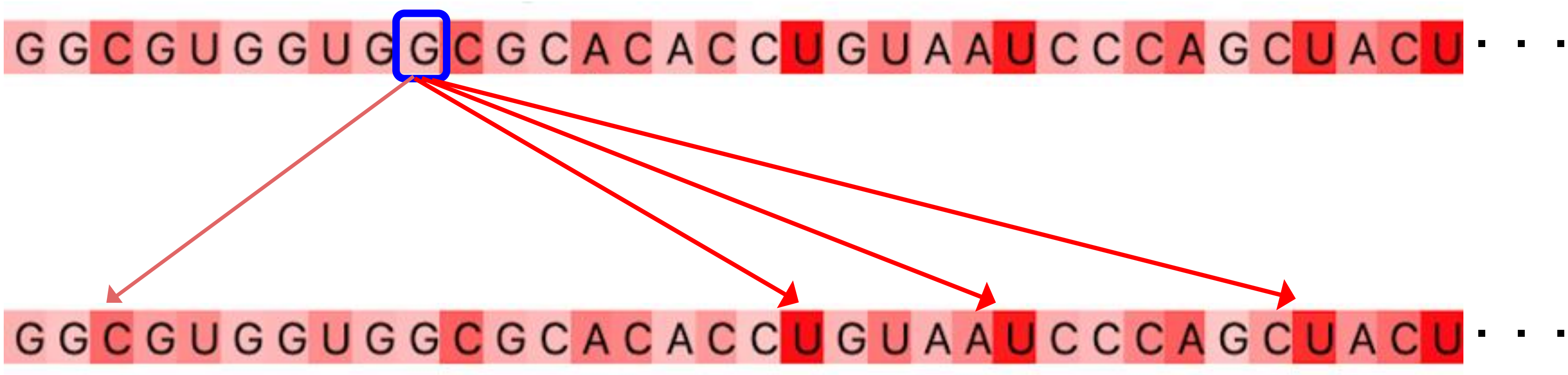
GGCGUGGUGGCGCACACCUGUAAUCCCCAGCUACU . . .
GGCGUGGUGGCGCACACCUGUAAUCCCCAGCUACU . . .

Supplemental Figure S3. Visualization of embedding of six secondary substructures with t-SNE; hairpin loop, base pair in stem, bulge and internal loop, multibranch loop, external loop at 3’, and external loop at 5’. The plot is displayed for each base.

Figure S3

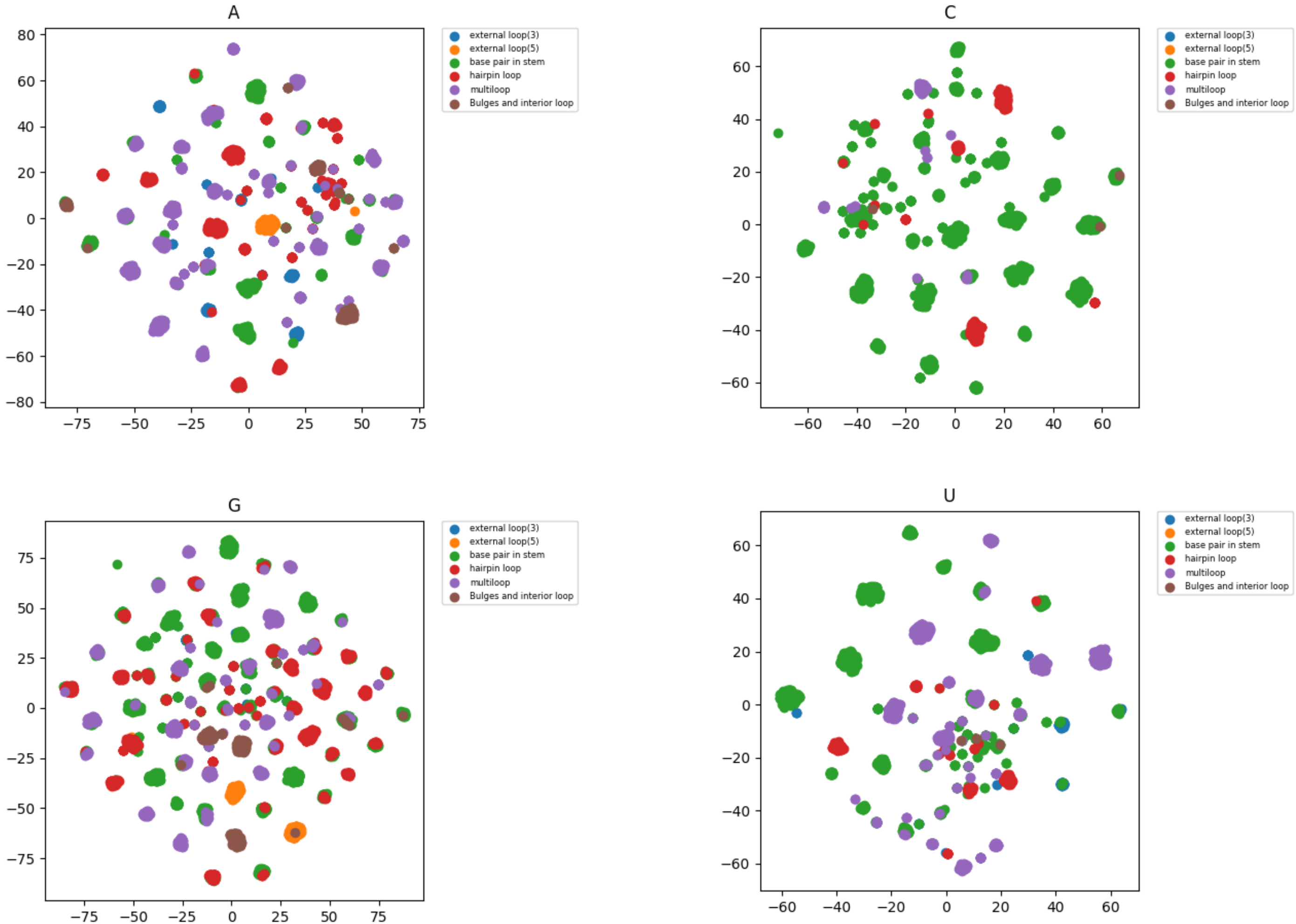
A
C
G
U
external loop(3)
external loop(5)
base pair in stem
hairpin loop
multiloop
Bulges and interior loop

Supplemental Figure S4. Illustration of six substructures in secondary structure of an RNA sequence.

Figure S4

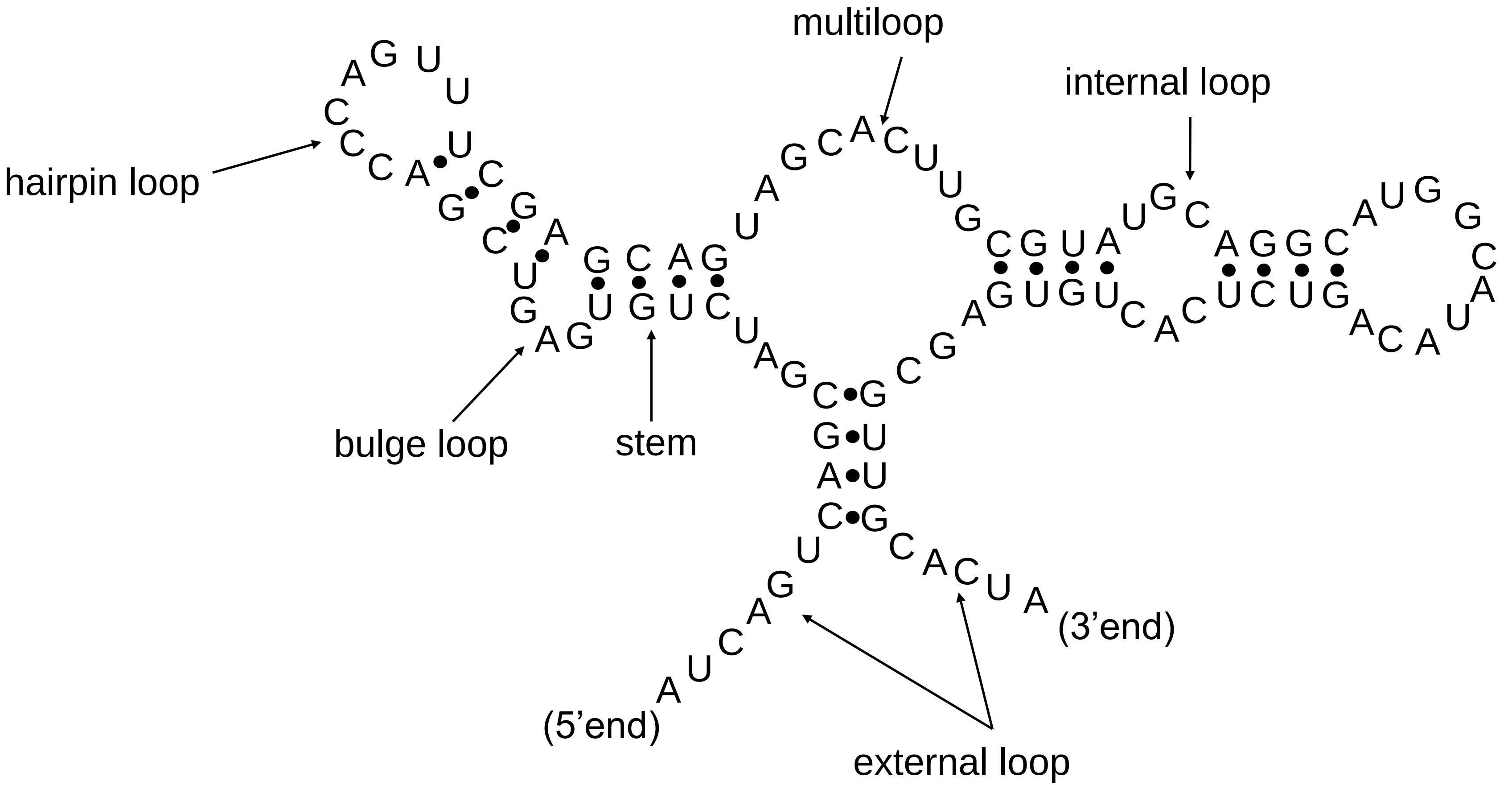

multiloop
internal loop
hairpin loop
bulge loop
stem
(5’end)
(3’end)
external loop
